# The complete mitochondrial genome of *Sorex minutissimus* (Eulipotyphla: Soricidae)

**DOI:** 10.1080/23802359.2019.1618207

**Published:** 2020-05-20

**Authors:** Bo Pang, Xingyao Chen, Dajie Xu, Xiufeng Yang, Weilai Sha, Honghai Zhang, Huashan Dou

**Affiliations:** a Hulunbuir Academy of Inland Lakes in Northern Cold & Arid Areas, Hulunbuir, P. R. China; b College of Life Science, Qufu Normal University, Qufu, P. R. China

**Keywords:** *Sorex minutissimus*, mitochondrial genome, phylogenetic tree

## Abstract

In this study, the complete mitochondrial genome of *Sorex minutissimus* was sequenced and deposited to GeneBank for the first time using muscle tissue. This mitochondrial genome is a circular molecule of 16,700 bp in length and sequence analysis showed it contains 2 rRNA genes, 22 tRNA genes, 13 protein-coding genes, rep_origin, and D_loop. Phylogenetic analysis on the basis of 12 protein-coding genes except *ND6* of 13 Soricidae species’ mitochondrial genomes using ML and BI demonstrated that *S. minutissimus* and other *Sorex* species were clustered into same clade.

The *Sorex minutissimus* is classified under order Eulipotyphla, family Soricidae and genus *Sorex*. It has a large range and habitats include forest-tundra zone, mixed forests, and forest-steppe and it feeds on small insects, spiders, grubs, and snails (Macdonald and Barrett 1993). In this study, the tissue sample of *S. minutissimus* was caught by pitfall traps through field survey and stored at biological specimens of Hulun Lake National Nature Reserve, Inner Mongolia, China, and the geo-spatial coordinates are 48°22′35″N latitude and 117°31′50″E longitude. All sampling procedures and experimental manipulations had the proper permits. After manually annotating, the mitochondrial genome was deposited in GeneBank with the accession number MK641805.

The complete mitochondrial genome of *Sorex minutissimus* is a double-circular DNA of 16,700 bp in length and contains 13 protein-coding genes, 22 tRNA genes, 16S rRNA, 12S rRNA, rep_origin, and D_loop. Among these genes, *ND6* and 8 tRNA (*tRNA^Asn^*, *tRNA^Ser^*, *tRNA^Ala^*, *tRNA^Glu^*, *tRNA^Cys^*, *tRNA^Tyr^*, *tRNA^Pro^*, and *tRNA^Gln^*) genes were encoded in L-strand and other genes were encoded in H-strand. The base composition is 33.0% for A, 28.9% for T, 13.3% for G, 24.8% for C and the percentage of A and T (61.9%) is higher than G and C (38.1%). This genes arrangement is similar to other species, such as *Sorex araneus* (Huang et al. 2016), *Sorex tundrensis* (Xu et al. 2015) and *Ochotona dauurica* (Yang et al. 2019).

Phylogenetic analysis of 9 *Sorex* species, 2 *Episoriculus* species, and 2 *Blarinella* species were analyzed using the maximum-likelihood (ML) and the Bayesian inference (BI) methods based on the 12 protein-coding genes except *ND6* with *Microtus arvalis* (NC_038176) used as an outgroup. Using MrModeltest 3.7 (Nylander 2004), GTR + I + G was selected as the best-fitting nucleotide substitution mode according to the AIC criterion. These parameters were used in BI and the ML analysis by MrBayes 3.2.2 (Ronquist and Huelsenbeck 2003) with a bootstrap test of 100 replicates and PAUP 4.0b10 (Swofford 2002) was run for 1,000,000 cycles, respectively.

Phylogenetic analysis shows that the different tree-building methods (ML and BI) have the same topology (Figure 1), and three major phyletic lineages were present in *Sorex*. The *S. minutissimus* was close to *S. sinalis*, *S. caecutien*s, *S. isodon*, and *S. gracillimus*, and had the greatest difference with *S. cylindricauda*. Compared with *Episoriculus*, the *Sorex* was close to *Blarinella* in the family of Soricidae, which was also supported by several previous studies (Jin et al. 2017). We expect the data of the present study to be useful for further research and for phylogenetic relationships of *Sorex*.

**Figure 1.**
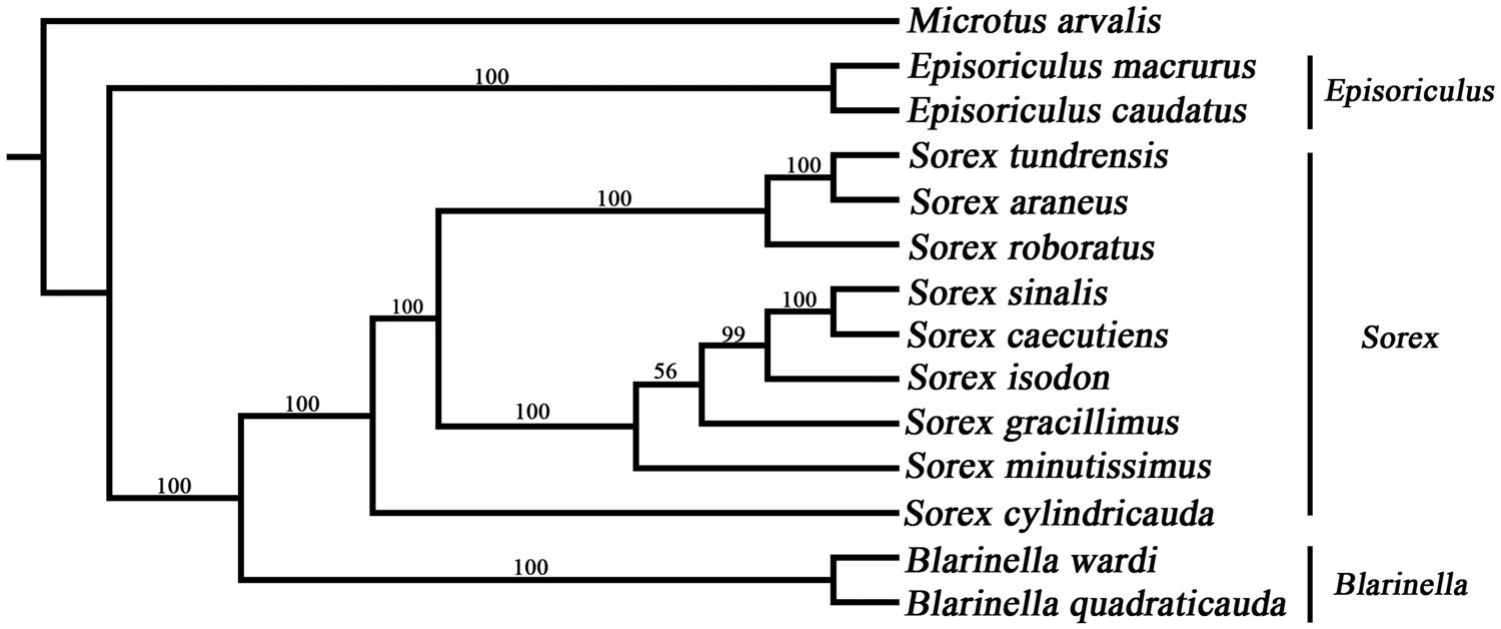
Phylogenetic tree of 13 species was obtained from maximum-likelihood (ML) and Bayesian phylogenetic inference (BI) methods, based on 12 protein-coding genes except *ND6* and the BI posterior probabilities are shown on the nodes. The species accession numbers were downloaded from GenBank and are as follows: *E. caudatus* (NC_026131), *E. macrurus* (NC_029840), *S. tundrensis* (NC_025327), *S. araneus* (NC_027963), *S. roboratus* (NC_034808), *S. sinalis* (NC_037174), *S. caecutiens* (MF374796), *S. isodon* (NC_037894), *S. gracillimus* (NC_037859), *S. cylindricauda* (KT023074), *B. quadraticauda* (NC_023950), *B. wardi* (MF125692) and *M. arvalis*.
